# Socioeconomic Factors Correlation With Idiopathic Scoliosis Curve Type and Cobb Angle Severity

**DOI:** 10.7759/cureus.34993

**Published:** 2023-02-14

**Authors:** Logan Laubach, Viraj Sharma, Abdulaziz Alsumait, Benjamin Chiang, Victoria Kuester

**Affiliations:** 1 Orthopaedic Surgery, Virginia Commonwealth University School of Medicine, Richmond, USA; 2 General Surgery, Riverside University Health System Medical Center, Riverside, USA

**Keywords:** food accessibility, insurance, financial difficulty, income, cobb angles, race, socioeconomic factors, idiopathic scoliosis

## Abstract

Introduction: Race and socioeconomic status correlate with disease outcomes and treatment in patients with idiopathic scoliosis (IS) to varying degrees, although there is no clear association with Cobb angle and curve type. The purpose of this study was to assess socioeconomic factors and their association with Cobb angles in patients with IS.

Methods: A retrospective chart review was completed with the radiographic analysis of 89 patients diagnosed with IS and spinal curves >10° between the ages of six and 18. Associations between the Cobb angles and socioeconomic categorical variables were analyzed using a nonparametric Kruskal-Wallis test and continuous variables using a Spearman Rank correlation.

Results: There were no significant associations between proximal thoracic, main thoracic, or thoracolumbar/lumbar Cobb angles and sex, insurance type, race, access to healthy food, financial difficulty, or income. BMI and proximal thoracic Cobb angle (ρ = 0.2375, p=0.0268) had a significant positive correlation, and BMI and income (ρ = -0.2468, p=0.0228) shared a significant negative correlation.

Conclusions: The severity of IS proximal thoracic Cobb angles was positively associated with BMI and income. Other socioeconomic factors such as age, race, sex, access to food, insurance, and financial difficulties related to scoliosis treatment were not correlated with Cobb angle severity. The data presented suggest that patients with IS have varying degrees of curve type and severity that overall do not correlate with various socioeconomic factors. Validating which factors are predictive of curve severity could lead to early intervention preventing further morbidity of IS.

## Introduction

Idiopathic scoliosis (IS) is prevalent in about one percent of the population and affects diverse patient populations [1,2]. Some genetic factors have been discovered to correlate with IS, although there is no definitive association [3]. The diversity of patients affected by a disease can have varying relations to their diagnosis, treatment, and outcomes. The relationship with race and other various socioeconomic factors has been shown to affect both the severity and prevalence of various disease processes such as trauma [4], myocardial infarction [5], and hypertension [6], as well as treatment, detection, and outcomes [7-10] for these common conditions. The correlation between race and IS spine curvature varies, with some studies showing Black patients tend to present with higher degrees of curvature than White patients [11], while others show no clear relationship between race and spinal curvature [12-14]. Race has also been shown to affect treatment options and hospital courses in patients with IS, with one study showing White patients were more likely to have spinal fusion surgery with lower mortality rates and be admitted to large teaching hospitals in comparison to other races [15].

Patients with Medicaid insurance and IS were more likely to be of non-White race and have more medical comorbidities, spinal fusions with more vertebral levels, and longer hospital stays than those with private insurance, although no difference in postoperative outcomes was noted [16,17]. Some studies have shown that patients with higher access to private insurance present for IS evaluation at a younger age than those with Medicaid, regardless of race [11]. However, the association between income and insurance type with Cobb angle severity is less clear [2,17]. Race and socioeconomic status correlate with disease outcomes and treatment in patients with IS to varying degrees, although there is no clear association with spinal curve type or Cobb angle severity [2,11,15,17]. 

Defining associations between race and other socioeconomic factors may help with earlier detection and improved treatment outcomes for patients with IS and alleviate some of the health and economic disparities seen with more advanced spinal curve progression. Our study aims to assess the association, if any, of socioeconomic and environmental factors, including race, sex, insurance type, income, access to healthy food, and experiencing financial difficulty with proximal thoracic, main thoracic, and thoracolumbar/lumbar Cobb angles in patients with IS.

## Materials and methods

Following Virginia Commonwealth University Human Research Protection Program/Institutional Review Board approval (HM20021605) at our institution for the study population, a retrospective chart review was completed with the radiographic analysis of patients diagnosed with IS and spinal curves >10° between the ages of six and 18. 623 patients were identified with ICD-10: M41.1, ICD-9: 737.30 codes for IS, and CPT codes for spinal fusion for scoliosis are 22800, 22802, 22804, 22840, 22842, and 22844. Patients’ X-rays were measured for Cobb angles in proximal thoracic, main thoracic, and thoracolumbar/lumbar regions of the spine using the Cobb method with a digital radiographic measurement tool [18-20]. Three hundred forty-three patients remained after excluding all patients with Cobb angles less than 10°. Patients with less than 10° Cobb angles were excluded. Of these patients, 89 had filled out the Scoliosis Research Society (SRS-30) forms for our final cohort analysis. Patients were classified using the original Lenke scoliosis classification system [21].

Socioeconomic factors were chosen based on previously published literature evaluating age, BMI, sex, insurance type, average income using zip code as a proxy, and self-reported race [2,11,15,17]. Scoliosis Research Society (SRS-30) forms were evaluated for reported financial difficulties in relation to their child’s idiopathic scoliosis. Patients’ charts were used to evaluate demographics such as age, BMI, sex, insurance type, and self-reported race. Patients’ zip code residence was used as a proxy for the average income for that region from the US Census Bureau [22]. Patients’ zip codes were also used to assess their access to food based on their residence using the Food Access Research Atlas through the Economic Research Service by the United States Department of Agriculture (USDA) [23]. Low access to healthy food is defined by the USDA as “Limited access to supermarkets, supercenters, grocery stores, or other sources of healthy and affordable food may make it harder for some people to eat a healthy diet in this country” [24]. Each zip code residence was entered into the Atlas to determine if each patient belonged to a low-income tract with limited access to healthy food, defined by the USDA as “at least 500 people, or 33 percent of the population, living more than one-half mile, or mile (urban areas) or more than 10 or 20 miles (rural areas) from the nearest supermarket, supercenter, or large grocery store.” and recorded.

Statistical analysis was supported by the Biostatistics Consulting Laboratory at our institution. Categorical data are summarized using frequency and percent, while continuous measures are summarized using number, mean, standard deviation, and 95% confidence intervals on the mean. For analysis purposes, several categorical variables were recorded due to small numbers. Both Race and Experiencing financial difficulties were reclassified into two levels; Race was reclassified as White/Caucasian, and Other while experiencing financial difficulty was reclassified as None vs. Any. Data from the Food Access Research Atlas were recoded into ordinal data for analysis as follows: no limited access to food= 0; 0.5 urban mile or 10 rural miles from food = 1; 1 urban mile or 10 rural miles from food= 2; 1 urban mile or 20 rural miles from food= 3. To assess associations between Cobb angles and the categorical demographic and socioeconomic variables (sex, insurance, race, financial difficulty, food accessibility), we utilized a non-parametric Kruskal-Wallis test. Associations between Cobb angles and continuous demographic and socioeconomic variables (age, BMI, income) were assessed using both a Spearman Rank correlation as well as a linear rank regression. JMP statistical software (version 15) was utilized for data analysis, and all analyses were performed at the α = 0.05 level of significance.

## Results

Of 89 patients, 74% were female, and 26% were male, with an average age of 12.3 and an average BMI of 21.6. 56% of patients identified as White/Caucasian, 35% as Black/African American, 3% as Asian/Pacific Islander, and 6% as other. 69% had private insurance, 31% had Medicaid, and none were uninsured. 42% underwent spinal fusion surgery. Eight percent of patients experienced financial difficulties as a result of their child's IS, and the average family income was $93,409.30 for their corresponding zip code region. 54% of patients did not live in an area with limited access to healthy food, 21% lived more than 0.5 urban miles or 10 rural miles from food, 7% 1 urban mile or 10 rural miles, and 18% more than 1 urban mile or 20 rural miles. The average proximal thoracic Cobb angle was 18.36° (standard deviation [SD] = 13.013°), the average main thoracic Cobb angle was 34.18° (SD = 19.216°), and the average thoracolumbar/lumbar Cobb angle was 27.10° (SD = 13.984°) (Tables 1, 2).

**Table 1:** Data Summary of Socioeconomic Categorical Variables

Categorical Variables	% (n)
Sex	
Female	74% (66)
Male	26% (23)
Insurance Type	
Private	69% (61)
Medicaid	31% (28)
Underwent Spinal Fusion Surgery	43%(38)
Race	
Black/African American	36% (32)
White/Caucasian	55% (49)
Asian/Pacific Islander	3% (3)
Other	6% (5)
Experienced Financial Difficulties	
None	92% (82)
Slightly	6% (5)
Moderately	2% (2)
Limitations to Accessible Food	
No	54% (48)
0.5 Urban or 10 Rural miles	21% (19)
1 Urban or 10 Rural miles	7% (6)
1 Urban or 20 Rural miles	18% (16)
Lenke Classification	
1	21% (19)
2	10% (9)
3	12% (11)
4	22% (20)
5	26% (23)
6	8% (7)

**Table 2:** Data Summary of Socioeconomic Continuous Variables BMI: Body mass index

Continuous Variables	n	Mean(SD)	95% CI
Age (years)	89	12.3 (2.09)	(11.8, 12.7)
BMI (kg/m^2^)	89	21.6 (5.8)	(20.4, 22.8)
Average Income ($)	89	93,409.3 (29,750.42)	(87,068.6, 99,750.0)
Coronal Cobb Angles (degrees)			
Proximal Thoracic	89	18.36 (13.013)	(15.62, 21.10)
Main Thoracic	89	34.18 (19.216)	(30.13, 38.23)
Thoracolumbar/Lumbar	89	27.10 (13.984)	(24.16, 30.05)

Proximal thoracic Cobb angle and sex (p=0.7111), insurance type (p=0.4473), race (p=0.8501), experiencing financial difficulties (p=0.3968), and limited access to healthy food (p=0.9331) were not statistically correlated. Thoracic Cobb angle and sex (p=0.7830), insurance type (p=0.6083), race (p=0.3555), experiencing financial difficulties (p=0.2497), and limited access to healthy food (p=0.5446) were not statistically correlated. Thoracolumbar/lumbar Cobb angle and sex (0.5465), insurance type (p=0.7205), race (p=0.3216), experiencing financial difficulties (p=0.3836), and limited access to healthy food (p=0.3565) were not statistically correlated (Table 3).

**Table 3:** Association of Cobb Angles With Socioeconomic Factors

	Proximal Thoracic Cobb Angle		Main Thoracic Cobb Angle		Thoracolumbar/Lumbar Cobb Angle	
	𝝌^2^	p-value	𝝌^2^	p-value	𝝌^2^	p-value
Sex	0.14	0.7111	0.08	0.7830	0.36	0.5465
Insurance	0.58	0.4473	0.26	0.6083	0.13	0.7205
Race	0.04	0.8501	0.85	0.3555	0.98	0.3216
Financial Difficulties	0.71	0.3968	1.32	0.2497	0.76	0.3836
Limited Access to Healthy Food	0.43	0.9331	2.14	0.5446	3.24	0.3565

There are statistically significant associations between BMI and Income (ρ = -0.2468, p=0.0228) and BMI and proximal thoracic Cobb angle (ρ = 0.2375, p=0.0268). No other statistically significant associations were seen between age, BMI, and average income with the proximal thoracic, main thoracic, and thoracolumbar/lumbar Cobb angle measurements (Table 4).

**Table 4:** Spearman Rank Correlations of Continuous Socioeconomic Variables and Cobb Angle

	Proximal Thoracic Angle		Main Thoracic Angle		Thoracolumbar/Lumbar Angle	
	Spearman’s ⍴	p-value	Spearman’s ⍴	p-value	Spearman’s ⍴	p-value
Age (years)	-0.0374	0.7282	0.0434	0.6862	0.1190	0.2665
BMI (kg/m^2^)	0.2375	0.0268	0.1182	0.2755	0.0294	0.7868
Income ($)	-0.0229	0.8830	-0.1072	0.3229	-0.1892	0.0792

## Discussion

The demographics of patients with IS have shown that females have a two-fold increase in incidence over males as well as different races having varying degrees of Cobb angle severity [1,13]. Our patient population aligns with the literature, as over twice as many patients were female, and a diverse patient cohort was present (Table 1). The association between race and ethnicity and the severity of the spinal curve severity as measured by Cobb angles varies greatly between studies. One study showed that Hispanic patients had lower Cobb angles in comparison to all other races, while other studies show that Black patients had the highest Cobb angles, and other studies further showed no difference in Cobb angle severity between races [11,13,25,26]. While no definite associations between race and curve severity can be concluded, there have been notable correlations between socioeconomic factors and race that may provide an explanation for the variability in the race and IS prevalence and curve severity.

Black patients with IS have been shown to have more limited health insurance plans, are more likely to have Medicaid, and have lower average incomes than other races [11]. Our results show there was no difference between race and insurance type, while Blacks had significantly lower average annual incomes compared to both Whites (p=0.003) and Asians (p=0.024) (data not shown). These disparities have led to Black patients presenting for diagnosis of IS at later ages with more severe spinal curve severities leading to them being more likely to have spinal fusion surgery and worse outcomes with increased mortality [4,11,15,26]. There was no significant difference in patients experiencing financial difficulty as a result of the treatments related to IS based on race or insurance type, although one study found that patients with IS on Medicaid had higher hospital costs than patients with private insurance [16]. 

There were no statistically different associations with sex, insurance type, experiencing financial difficulties related to IS, race, or income, and proximal thoracic, main thoracic, or thoracolumbar/lumbar Cobb angle severity in our study (Tables 3, 4). This aligns with current literature, with these economic variables having no significant relationship with Cobb angle severity [2,11,17]. While certain races are more likely to present with higher curve severities, lower socioeconomic status, and increased mortality, there does not appear to be a direct relationship between Cobb angle severity and socioeconomic variables.

BMI and proximal thoracic Cobb angle (ρ = 0.2375) had a significant positive correlation but no correlation with the main thoracic or thoracolumbar/lumbar Cobb angles (Table 4). The linear rank regression indicates that while the association between BMI and the Cobb proximal thoracic angle is statistically significant, BMI only explains 5% of the variability in the proximal thoracic Cobb angle. BMI has different associations with IS curve severity; some studies showing lower BMI is associated with higher Cobb angles [14,25], while others show a positive correlation between higher BMI and higher Cobb angles [27] as well as increased rates of inpatient and intraoperative complications related to IS [16,28,29]. Despite the variability in which BMI affects curve severity, both extremes in the literature suggest a correlation with increased Cobb angle and increased complication rates, which brings up nutrition as a possible contributing factor to BMI.

Our results show a significant negative correlation between BMI and income (ρ = -0.2468), showing that the lower the income, the higher the BMI, which has implications with increased Cobb angle and surgical complications, as mentioned previously. Interestingly we found no significant correlations between Cobb angle and patients living in “food deserts" being more than one urban or 20 rural miles from the nearest grocery store with access to fresh healthy food (Table 3). Food deserts themselves have been positively correlated with increased incidence of obesity, given the lack of access to healthy foods as well as the higher price for healthy foods [30-32]. The convenience and cheaper cost of unhealthy foods further perpetuate the likelihood of obesity living in these food deserts. Patients with IS and obesity were more likely to live in lower socioeconomic geographical locations and had higher readmission rates and wound dehiscence after spinal fusion for IS [29]. Additionally, poor nutrition has been associated with a higher incidence of IS [33], although more specifically, vitamin D deficiency and osteoporosis have been a topic of discrepancy in whether or not a correlation exists in patients with IS [34,35]. Our results indicate that while we found a negative correlation between BMI and income, there were no significant correlations between Cobb angle severity and limited access to healthy food. To our knowledge, no study to date has examined the relationship of food deserts to Cobb angle severity in patients with IS.

Our study limitations are a retrospective study at a single institution with a lack of randomization and a small sample size limiting the statistical power of possible clinical and socioeconomic differences. Our study was underpowered with the number of patients in both Asian/Pacific Islander as well as Other and Hispanic race categories to be able to show a statistically meaningful difference in factors such as clinically worse outcomes or likelihood of undergoing surgery for IS. Assessing income based on geographical location has limitations as it uses an average income for that zip code location. Given the lack of actual reported income in our patient database, we believe it is the most accurate way of assessing income, and many epidemiological studies have validated the use of zip code as a proxy for average income. Reporting bias was likely present in our study regarding if patients experienced financial difficulty utilizing the SRS-30 surveys, although this survey has been validated against other surveys to assess the quality-of-life measurements [36,37]. A larger follow-up study utilizing a larger sample size through either a multi-institution or open database analysis would provide further power to detect a correlation between IS Cobb angle severity and various socioeconomic factors if they exist.

## Conclusions

The severity of IS proximal thoracic Cobb angles was positively associated with BMI and income. Other socioeconomic factors such as age, race, sex, access to food, insurance, and financial difficulties related to scoliosis treatment were not correlated with Cobb angle severity. The data presented suggest that patients with IS have varying degrees of curve type and severity that do not correlate with various socioeconomic factors, which aligns with current literature. Further research is needed to validate correlations between BMI, income, and access to healthy food and their effects, if any, on Idiopathic Scoliosis. This would further help elucidate the possible predictive factors of which patients might be at risk for not only developing IS but also curve progression necessitating the need for early intervention to prevent further morbidity of IS.
